# Developmental Expression of Translocator Protein/Peripheral Benzodiazepine Receptor in Reproductive Tissues

**DOI:** 10.1371/journal.pone.0074509

**Published:** 2013-09-05

**Authors:** Kanako Morohaku, Newton S. Phuong, Vimal Selvaraj

**Affiliations:** Department of Animal Science, Cornell University, Ithaca, New York, United States of America; University of Nevada School of Medicine, United States of America

## Abstract

Translocator protein (TSPO) present in the outer mitochondrial membrane has been suggested to be critical for cholesterol import, a rate-limiting step for steroid hormone biosynthesis. Despite the importance of steroidogenesis in regulating reproductive functions, the developmental profile of TSPO expression in the gonads and accessory sex organs has not been completely characterized. As a first step towards understanding the function of TSPO, we studied its expression in male and female murine reproductive organs. We examined testes and ovaries at embryonic days 14.5 and 18.5, and postnatal days 0, 7, 14, 21 and 56 of development. In the adult testis, TSPO was expressed in both Leydig cells and Sertoli cells. In the developing testes TSPO expression was seen in immature Sertoli cells, fetal Leydig cells and gonocytes. In the ovary, TSPO was expressed in the ovarian surface epithelium, interstitial cells granulosa cells and luteal cells. Corpora lutea of ovaries from pregnant mice showed strong expression of TSPO. In the developing ovary, TSPO expression was seen in the squamous pregranulosa cells associated with germ line cysts, together with progressively increasing expression in interstitial cells and the ovarian surface epithelium. In adult mice, the epithelia of other reproductive tissues like the epididymis, prostate, seminal vesicle, oviduct and uterus also showed distinct patterns of TSPO expression. In summary, TSPO expression in both male and female reproductive tissues was not only restricted to steroidogenic cells. Expression in Sertoli cells, ovarian surface epithelium, efferent ductal epithelium, prostatic epithelium, seminal vesiclular epithelium, uterine epithelium and oviductal epithelium suggest either previously unknown sites for *de novo* steroidogenesis or functions for TSPO distinct from its well-studied role in steroid hormone production.

## Introduction

Translocator protein (TSPO) was first identified as a pharmacologically distinct diazepam-binding protein [1], [2], and has long been studied under its former name, peripheral-type benzodiazepine receptor (PBR) [3]. Biochemical characterization of this 18 kDa transmembrane protein showed predominant presence in the mitochondria, with specific localization to the mitochondrial outer membrane [4], [5]. Although it is highly conserved from bacteria to humans [6], the precise function of TSPO/PBR continues to remain elusive as evidence that is quite multifaceted points to several physiological and pathological roles for this protein (reviewed in [7], [8]). Experimentation on TSPO function has suggested involvement in cell proliferation [9], [10], apoptosis [11], [12], cellular respiration [13], heme synthesis [14], erythropoiesis [15], calcium flow [16], [17], cellular immunity [18], stress responses [19], photosensitization [20], malignancy [21], [22], and steroid hormone biosynthesis [23], [24]. Furthermore, its importance in vital functions was highlighted by embryonic lethality that was observed in TSPO gene deleted mice [25].

TSPO gene expression is regulated by several mechanisms in tissues and is not completely defined. Transcription factors such as specificity protein 1/specificity protein 3 (Sp1/Sp3) [26], activator protein 1 (AP1) [27], and v-ets erythroblastosis virus E26 oncogene homolog (Ets) [28], that act on the TSPO promoter have been linked to expression levels. More recently, a mechanism of regulation of TSPO expression by a natural antisense transcript called short interspersed repetitive element B2 (SINE B2) has been shown to regulate TSPO transcription [29].

Studies examining the conserved molecular structure of TSPO showed a channel-like conformation for this protein with five transmembrane alpha helices and a hydrophobic core [30]. A cholesterol recognition amino acid consensus (CRAC) has been characterized at its cytosolic carboxyl terminus suggesting cholesterol binding [31]. In the mitochondria, two specific proteins, voltage-dependent anion channel (VDAC) and the adenine nucleotide transporter (ANT) have been shown to interact with TSPO, suggesting existence of this protein as a complex [32]. This association with VDAC and ANT connected TSPO to being part of the mitochondrial permeability transition pore (MPTP), linking it to potential functions including initiation of the mitochondrial apoptosis pathway [33]. However, its interaction with VDAC and ANT was not found required for other TSPO functions like its role in steroid hormone production [34].

Specific function for TSPO in cholesterol transport required for steroidogenesis is perhaps the most characterized activity for this protein [35]. It was first identified that pharmacological agents Ro5–4864 and PK11195 that bind to TSPO modulated testosterone production in testicular Leydig cells [36], [37], and progesterone production in ovarian granulosa cells [38]. It was subsequently demonstrated that disruption of PBR/TSPO abolished steroid hormone biosynthesis in the R2C Leydig cell line [23]. Similarly, an antisense knockdown of PBR/TSPO expression in the MA-10 Leydig cell line also decreased steroid hormone biosynthesis [24].

Cholesterol transport to the inner mitochondrial membrane is essential to execute the first and rate-limiting step of the steroid hormone biosynthetic pathway [39]. In this step, mitochondrial P450 side chain cleavage enzyme (CYP11A) gains access to cholesterol at the inner mitochondrial membrane and catalyzes its conversion to pregnenolone [40]. To arrive at the inner mitochondrial membrane, cholesterol must traverse the aqueous space that lies between the outer and inner mitochondrial membranes. Involvement of the steroidogenic acute regulatory protein (StAR) in this function has been well established; mutations to the StAR gene results in lipoid congenital adrenal hyperplasia, a cholesterol transport disorder that results in impaired gonadal and adrenal steroid hormone biosynthesis [41], [42]. However, the exact steps involved in this transport, and parts played by other cooperating functional proteins remains an area of active investigation [43], [44]. Aligned with their functional similarities, there is strong accumulating evidence for a working cooperation between StAR and TSPO in steroid hormone biosynthesis [45], [46], [47].

Despite the potential importance of TSPO in regulating steroid hormone production for reproductive development and function, there have been no studies examining TSPO expression and localization exclusively focused on the reproductive system. In this study, we systematically examine the developmental expression and specific localization of TSPO in the testis, ovary and other reproductive tissues in the murine model.

## Materials and Methods

### Reagents

All reagents and chemicals were purchased from Sigma (St. Louis, MO), unless otherwise noted. A commercial rabbit monoclonal antibody against TSPO (Epitomics, Burlingame, CA) was used for all the localization studies and western blots. Specificity of this antibody was confirmed by blocking with the immunizing peptide (Epitomics). A mouse monoclonal antibody was used for localization of VDAC1 (Abcam, Cambridge, MA). For immunohistochemistry, a goat-anti-rabbit secondary antibody conjugated with polymerized horseradish peroxidase (pHRP) was used for diaminobenzidine (DAB)-based chemistry. Fluorescent goat anti-rabbit secondary antibody and goat anti-mouse Fab fragments were used for immunofluorescence (Jackson Immunoresearch, West Grove, PA). A micro BCA protein assay kit (Thermo Scientific, Rockford, IL) was used to quantify total protein. A mouse anti-actin polyclonal antibody (Li-Cor, Lincoln, NE) was used to estimate loading normalization in western blots. Species-specific fluorescent secondary antibodies (Li-Cor) were used to detect TSPO and actin in western blots. MA-10 Leydig cell line [48], was a generous gift from Dr. Mario Ascoli, Department of Pharmacology, The University of Iowa.

### Mice

Mice (C57BL/6J) were purchased from the Jackson Laboratories (Bar Harbor, ME), and were bred for the different experiments. Timed pregnancies were setup by checking for vaginal plugs to confirm mating. Gonads were collected from fetuses at embryonic stages (E14.5 and E18.5). Gonads were also collected from postnatal pups to adult mice at different ages (P0, P7, P14, P21 and P56/adult). In adult females, oviducts and uteri were also included in the collection. In adult males, the seminal vesicles and prostates were also collected. At least four animals from each sex were collected and examined for all the different time points. All animals were maintained in accordance to the National Institutes of Health Guidelines for the Care and Use of Laboratory Animals, and all experiments were performed with an approved protocol (# 2010-0098) from the Institutional Animal Care and Use Committee of Cornell University.

### Western Blots

To evaluate the specificity of the monoclonal TSPO antibody, we examined western blots for specific detection of an 18-kDa band representing TSPO. Freshly collected MA-10 cell samples were sonicated and boiled in Laemmli sample buffer, and total protein was quantified using a bicinchoninic acid colorimetric assay. Twenty micrograms of protein was then separated by SDS-PAGE, transferred to PVDF membranes and immunoblotted for the presence of TSPO. In brief, membranes were blocked using 5% non-fat dry milk in tris buffered saline containing 0.2% Tween 20 (TBST) and incubated with rabbit anti-TSPO monoclonal antibody and control mouse anti-actin affinity purified polyclonal antibody. For testing the specificity of the TSPO antibody, a preadsorption control was performed treating the antibody with a 10-fold molar excess of the TSPO immunizing peptide before use. Detection was performed by incubation with IRDye 800 conjugated goat anti-rabbit IgG and IRDye 700 conjugated goat anti-mouse IgG followed by imaging using a laser fluorescence scanner (Li-Cor) for simultaneous detection of the two emission wavelengths.

### Immunohistochemistry

Tissues were fixed with 4% formaldehyde in phosphate buffered saline for 48 hours at room temperature. Specimens were then processed, embedded in paraffin and 4 µm thin sections were prepared on glass slides. After deparrafinization and rehydration, sections were subjected to antigen retrieval using 0.01 M citrate buffer. Non-specific binding was blocked using 5% normal goat serum, then samples were incubated with anti-TSPO antibody (1∶200) in 1% BSA in PBS overnight at 4°C. For testing the specificity of the TSPO antibody, a preadsorption control was performed treating the antibody with a 10-fold molar excess of the TSPO immunizing peptide before incubation with the samples. After incubation, slides were washed in PBS and incubated with pHRP-conjugated anti-rabbit secondary antibody and processed using the DAB chemistry to visualize positive staining. To highlight morphology, slides were counterstained for a weak hematoxylin background. Images were acquired in a DM1000LED Leica microscope using an ICC50HD camera.

### Immunofluorescence

Leydig cells (MA-10) grown on coverslips were fixed with 4% formaldehyde and permeabilized using 0.1% Triton X-100. Cells were then blocked using 5% normal goat serum and incubated with anti-TSPO antibody (1∶200) and anti-VDAC1 antibody (1∶500). Cells were subsequently washed with PBS and incubated with Alexa Fluor conjugated anti-mouse Fab fragments (488 nm) and anti-rabbit antibody (555 nm) and then washed again, counterstained with DAPI and mounted using an antifade reagent. Testis sections prepared as described above were also stained using anti-TSPO and anti-VDAC1 antibody using primary antibody dilutions as specified for MA-10 cells. An additional blocking step with unlabeled anti-mouse Fab fragments (13 µg/ml) was included before primary antibody incubation to prevent non-specific recognition of endogenous IgG when using the anti-mouse secondary antibody on tissues. Stained MA-10 cells and testis sections were imaged using a Zeiss LSD510 confocal microscope.

## Results and Discussion

Steroid hormones play a critical role in the development and function of both male and female reproductive organs. With advances in understanding of steroid hormone biosynthesis in a cell [49], studies on the cell type specific expression of proteins in reproductive tissues have become critical to complement and reinforce any functional interpretations. In the case of proteins like TSPO that are widely expressed and known to be involved in more than one function [7], [50], specific *in vivo* observations can lead to not only a better comprehension, but also novel insights into physiological mechanisms. In this study, we characterize the developmental and functional expression of TSPO, to better comprehend its role in different reproductive tissues and steroidogenesis. These findings in the reproductive system also translate to functional understanding of TSPO in other organ systems and its basis for expression in specific pathologies that include ischemic stroke, neuroinflammation and certain tumors.

**Figure 1:**
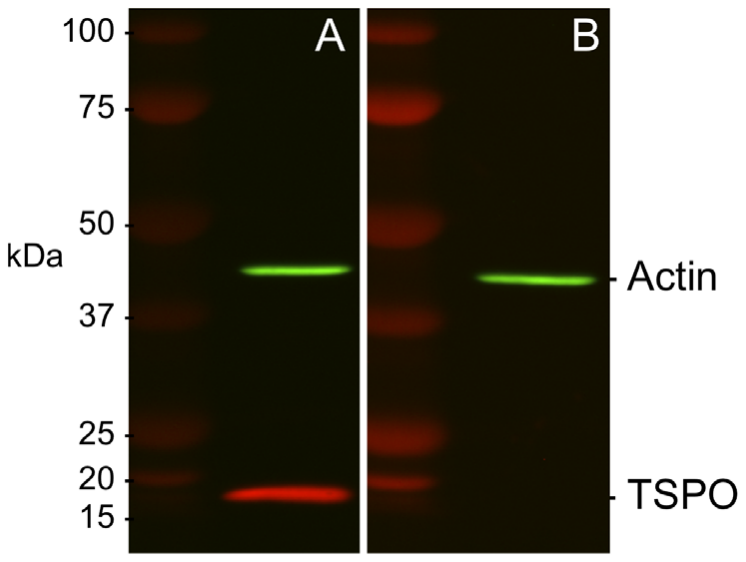
TSPO antibody is highly specific. (A) Western blot showing specific recognition of TSPO in MA-10 cell lysates as a single 18-kDa band by the rabbit monoclonal anti-TSPO antibody that was used for all experiments in this study. (B) Peptide-preadsorbed control for the same sample does not show this band confirming the specificity of the antibody in recognizing TSPO. Beta-actin was used as a loading control for both blots as indicated.

**Figure 2:**
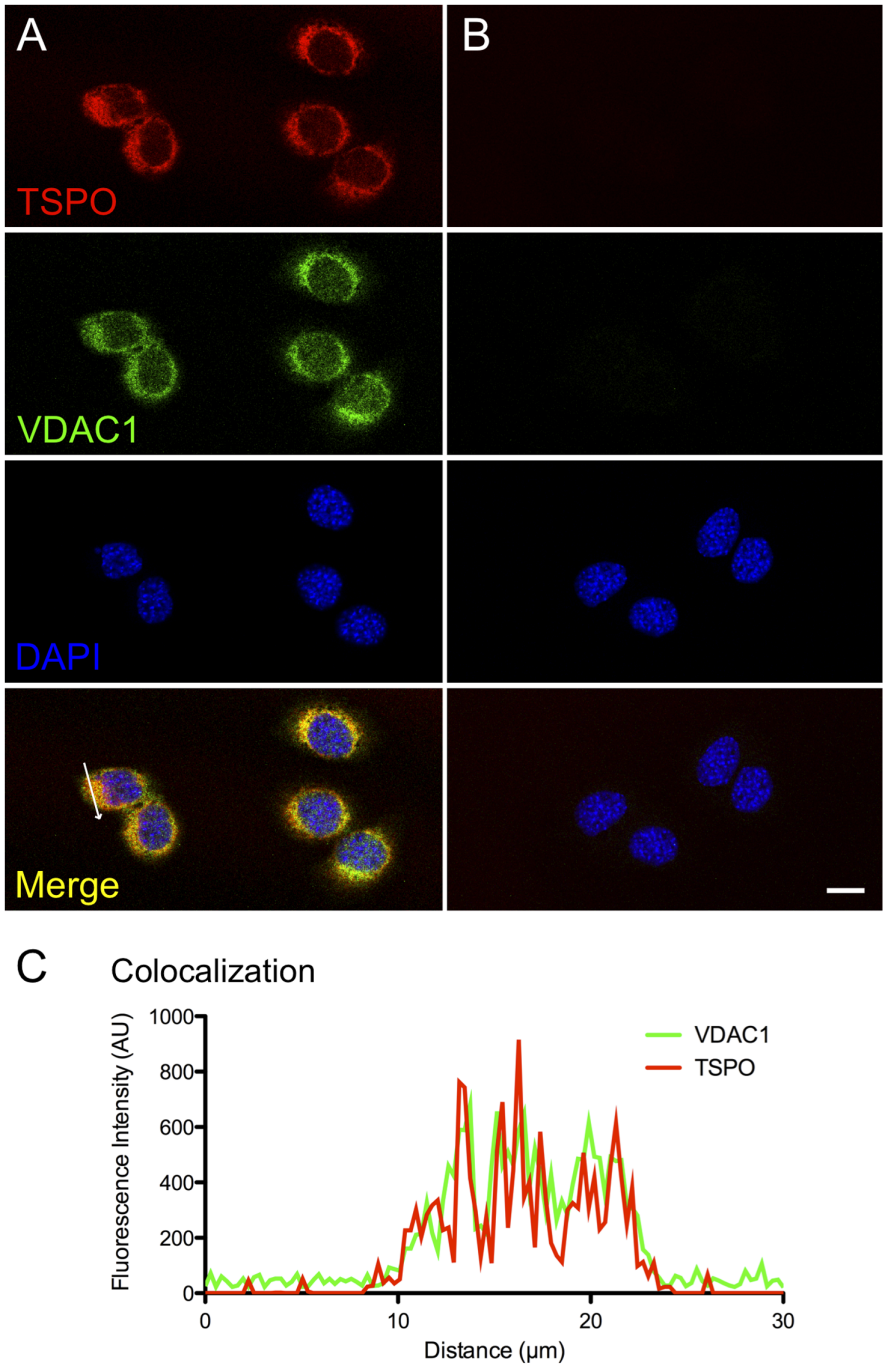
TSPO expression is localized to the mitochondria. (A) Panel shows confocal images of TSPO (red), VDAC1 (green) and nuclear counterstain (blue) in MA-10 Leydig cells. (B) Negative control panel. (C) Graph shows fluorescence intensities for TSPO and VDAC1 across the region indicated by a white arrow in Panel A. Colocalization of TSPO to the mitochondrial protein VDAC1 validates the specific localization of TSPO. Scale bar 20 µm.

**Figure 3:**
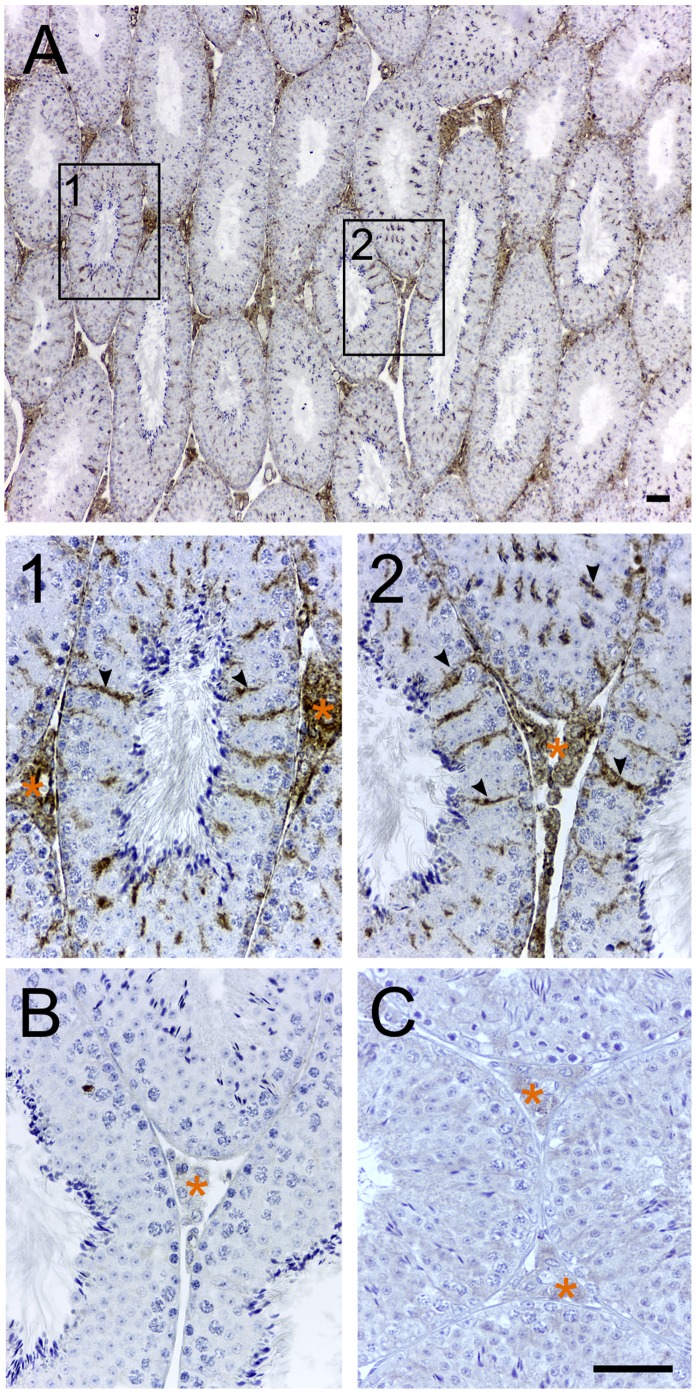
TSPO expression in the adult testis is localized to Leydig and Sertoli cells. Immunohistochemical localization of TSPO in testes from an 8-week-old mouse. (A) Prominent TSPO expression was observed in the interstitial Leydig cells. Boxed regions 1 and 2 within Panel A under higher magnification showed specific TSPO expression in Sertoli cells (few indicated with arrowheads) in addition to Leydig cells (region with orange asterisk). (B) Negative control without the monoclonal primary antibody did not show any labeling. (C) Peptide-preadsorbed control did not show any labeling validating the specificity of this antibody for detecting TSPO in immunohistochemical sections. Scale bars 20 µm.

In early studies, radiolabelled lipophilic chemicals [^3^H]Ro5–4864 [6], or [^3^H]PK11195 [51] that bind TSPO have been used to roughly evaluate expression by exposing treated tissue sections to radiographic film [51]. Polyclonal anti-sera that were generated against TSPO till date have largely lacked the specificity required for immunolocalization experiments. The rabbit monoclonal TSPO primary antibody that was used for all immunolocalization experiments in this study was first validated for specific recognition of TSPO protein. In western blots from protein lysates from a Leydig cell line (MA-10), the rabbit monoclonal TSPO antibody recognized a single 18 kDa band that corresponds to the expected molecular weight defined by the murine TSPO cDNA sequence [52]; this TSPO band disappeared in the peptide-preadsorbed control showing the specificity of this antibody in recognizing TSPO (Fig. 1). Using immunofluorescence and confocal imaging, we also confirmed that TSPO fluorescence was associated with the mitochondria. TSPO fluorescence in MA-10 cells colocalized with the mitochondrial marker VDAC1 (Fig. 2). In immunohistochemistry, specific recognition of TSPO was confirmed by the absence of labeling in the peptide-preadsorbed control (Fig. 3C). Therefore, the epitope recognized by this monoclonal antibody was very specific for TSPO protein (these control experiments were repeated three times). The specificity of this reagent allowed us to perform all experiments with precision, and reliably localize TSPO in various reproductive tissues.

### TSPO Expression in the Adult Testis

**Figure 4:**
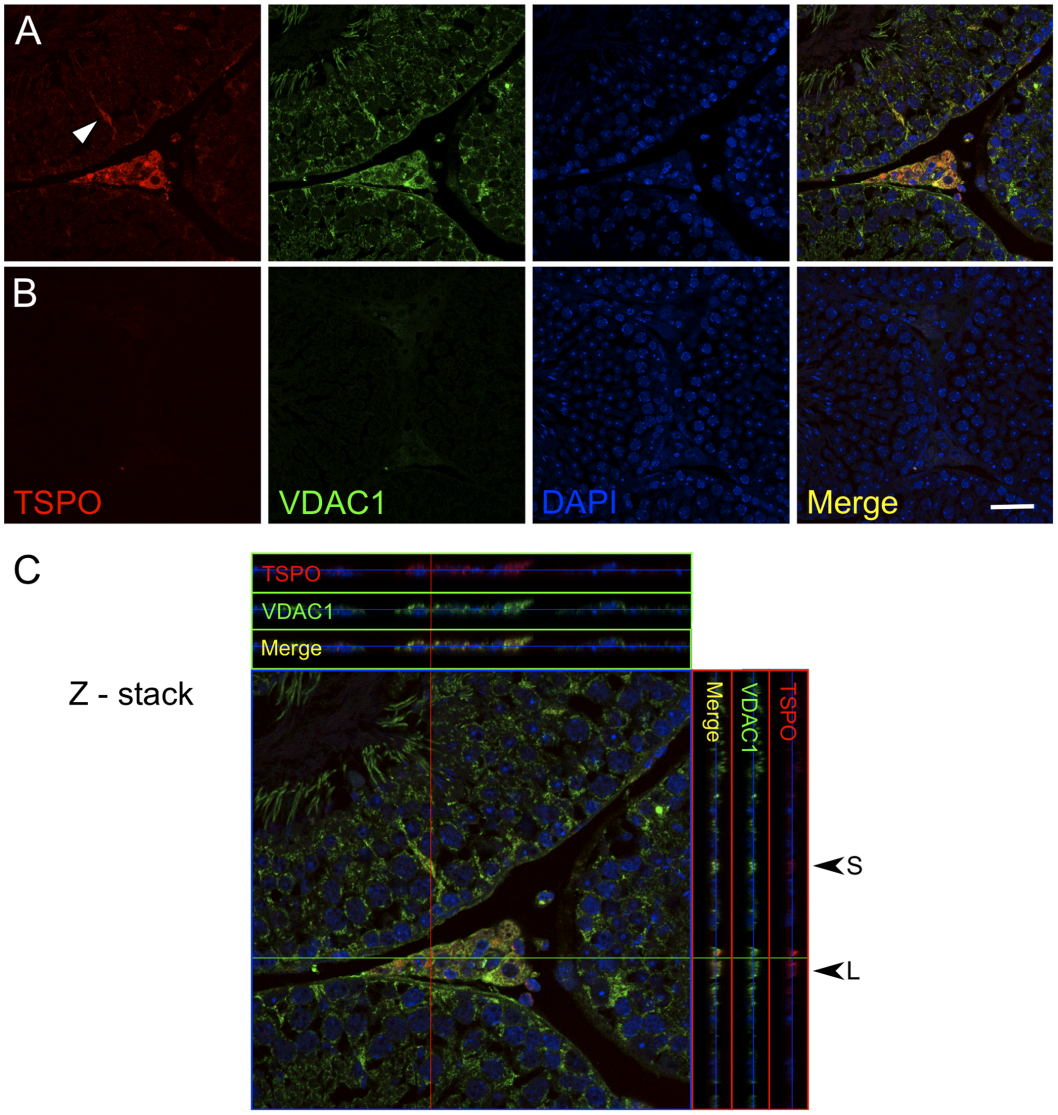
TSPO colocalizes with VDAC1 in Leydig and Sertoli cells. (A) Panel shows confocal images of TSPO and VDAC1 localization in the testis. TSPO labeling is not apparent in germ cells but specific fluorescence is visible in Sertoli cells (arrowhead) and Leydig cells. VDAC1 localization is seen in all cellular mitochondria including developing germ cells and the midpiece of spermatozoa. TSPO colocalization with VDAC1 is seen specifically in Leydig and Sertoli cells. (B) Negative control panel. (C) A 21-image Z-stack shows colocalization of TSPO and VDAC1 across optical sections in the X (green line) and Y (red line) axes. Specific overlap is seen at regions representing Leydig cells (L) and Sertoli cells (S). Scale bar 20 µm.

The first study to detect TSPO localization in the testis utilized [^3^H]Ro5–4864 as a tracer and showed high binding in interstitial regions between the seminiferous tubules [6]. In this study, we observe a specific localization of TSPO to not only the interstitial Leydig cells, but also to the Sertoli cells within the seminiferous tubules (Fig. 3). TSPO expression was not apparent in spermatogonial stem cells or in the developing germ cells at different stages of the seminiferous tubules. This observation was corroborated by immunofluorescence confocal detection in testes sections that showed TSPO and VDAC1 colocalization in Leydig and Sertoli cells (Fig. 4). TSPO expression was not evident in VDAC1-labelled mitochondria at the different stages of germ cells in the seminiferous tubules.

Leydig cells are the steroidogenic cells in the testis that produce testosterone. Based on the known function for TSPO in cholesterol transport across the mitochondrial membranes, expression of TSPO along with StAR [53] and other steroidogenic enzymes is expected for functional Leydig cells [54]. However, in adult Sertoli cells, there is little evidence to support a role for TSPO in steroidogenesis. One can just speculate that it may perhaps be involved in cholesterol transport into the mitochondria for other modifications. Although a study examining human testis showed that StAR is expressed in Sertoli cells [55], evidence for the expression of CYP11A1 (P450 side chain cleavage) that converts cholesterol to pregnenolone in Sertoli cells remains controversial [56]. The conversion of cholesterol to pregnenolone was initially suggested to be possible within seminiferous tubules [57]; however, this observation has been refuted by several subsequent studies that have questioned Sertoli cell potential for *de novo* steroidogenesis [58], [59], [60]. Therefore, the function of TSPO in adult Sertoli cells remains unclear.

### TSPO Expression in the Developing Testis

**Figure 5:**
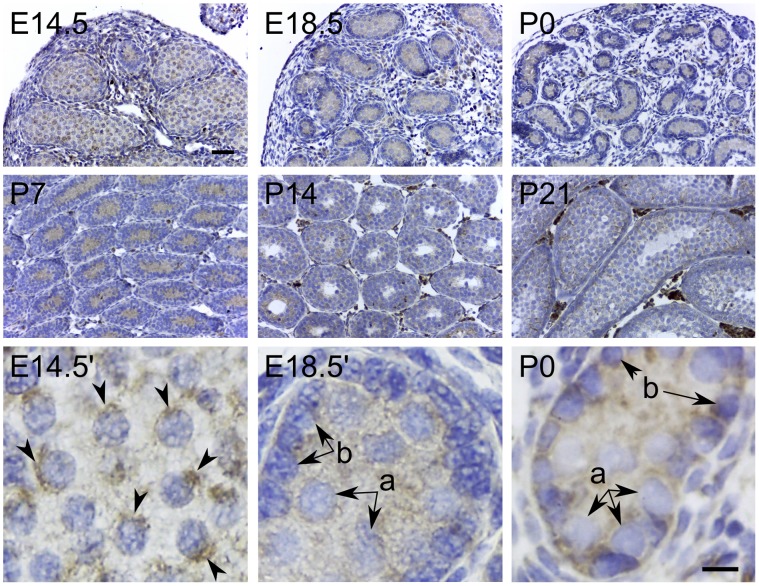
Developmental expression of TSPO in the testis. Immunohistochemical localization of TSPO in embryonic (E14.5 and E18.5), and early postnatal stages (P0, P7, P14 and P21) of the developing testis. At stages E14.5, E18.5, P0 and P7, TSPO was diffusely expressed within the seminiferous cords in both gonocytes and Sertoli cells; few strongly positive cells were also seen among the interstitial cells. At E14.5 TSPO localization in gonocytes was seen enriched in a perinuclear compartment (E14.5′ arrowheads). In higher magnification images from E18.5 and P0, TSPO localization persisted weakly in both gonocytes and proliferating Sertoli cells (E18.5′ and P0′; a - gonocytes, b – Sertoli cells). At P14 and P21, TSPO expression was approaching a pattern similar to the adult testis. However, Leydig cell expression levels at these age groups appeared higher compared to the adult (compare Fig. 2). Scale bars 20 µm.

Fetal testosterone production by the testis is a critical regulator of sexual differentiation [54]. During embryonic development, it has been reported that CYP11A1 can be detected as early as E12.5 in the fetal testis [61]. Other major enzymes required for androgen biosynthesis (CYP17A1 and 3βHSD) have been detected by E13 [62], the time point immediately after sexual differentiation to the male phenotype occurs (E12). When we examined the embryonic testis, we observed weak TSPO expression within the seminiferous cords along with a few strongly positive interstitial cells at E14.5 moderately increasing in intensity to P7 testis (Fig. 5). At E14.5, TSPO expression was localized to defined juxtanuclear regions within the gonocytes. At E18.5, weak TSPO expression was seen in both the immature Sertoli cells and gonocytes. This expression in gonocytes persisted postnatally until P7. From P14 to P21, expression in the fetal proliferating Leydig cells became more prominent. The role of fetal Leydig cells in androgen production is well documented [63]. Within the tubules, TSPO expression at P21 closely resembled the pattern of expression found in the adult testis. Fetal TSPO expression in Sertoli cells could be associated with steroid hormone biosynthesis. In rats, immature Sertoli cells in the fetus are known to express CYP11A1 when cultured *in vitro* with follicle stimulating hormone, and can produce pregnenolone [64]. However, TSPO expression in gonocytes, and not in adult stages of germ cell development (including spermatogonial stem cells) suggest a stage-specific developmental function. A recent study examining TSPO in rat testicular germ cells reported expression in both gonocytes and adult germ cells [65]. Using a polyclonal antibody, they showed that TSPO was predominantly localized to the nucleus. In this study, we find TSPO localized to juxtanuclear regions in gonocytes (Fig. 5), but expression was not apparent in adult germ cells (Fig. 3 and Fig. 4). This dissimilarity identifies an interesting species difference between mouse and rat testicular germ cells. Similar differences in expression between mouse and rat germ cells have been reported for other genes [66].

### TSPO Expression in the Epididymis

**Figure 6:**
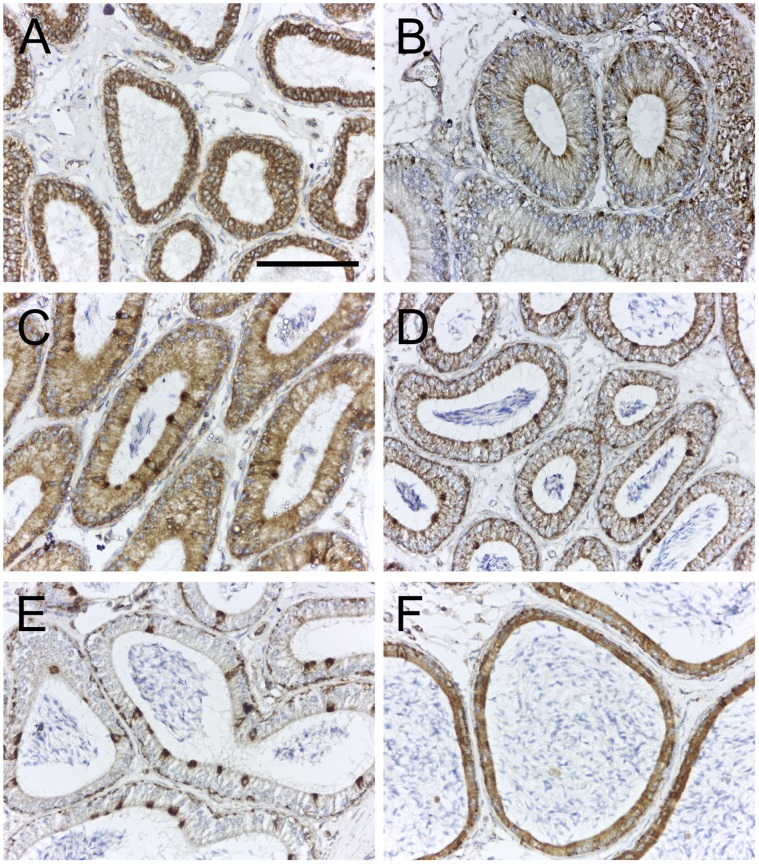
TSPO is expressed in the epithelium of the efferent ducts and epididymis. Immunohistochemical localization of TSPO in efferent ducts and segments of the epididymis from an 8-week-old mouse. (A) TSPO expression in the epithelium of the distal efferent ducts; all cells forming the epithelial layer showed strong expression of TSPO. (B) TSPO expression in the epithelium of segment I of the caput epididymis; expression was confined to the apical and basal regions, specific to narrow and basal cells. (C) TSPO expression in the epithelium of segment II of the caput epididymis; all cells expressed TSPO but prominently higher expression was observed in the narrow cells. (D) TSPO expression in the epithelium of segment III of the caput epididymis; expression was weaker than that observed in segment II, but the levels in narrow cells and basal cells appeared unchanged. (E) TSPO expression in epithelium of the corpus epididymis; localization was restricted exclusively to the basal cells and clear cells. (F) TSPO expression in epithelium of the cauda epididymis; almost all cells forming the epithelial layer appeared to show TSPO expression. Scale bars 20 µm.

It is well established that the post-testicular maturation of spermatozoa occurs starting at the efferent ducts and extending through the length of the epididymis. However, understanding of functions mediated by the epididymal epithelium remains fairly limited [67]. In this study we find that TSPO is expressed in the epithelium of the distal efferent ducts, and in the different segments of the caput, corpus and cauda epididymis (Fig. 6). Among the different cell types forming the epididymal epithelium, TSPO expression was distinctly prominent in narrow cells, clear cells and basal cells; expression in principal cells was weaker and also varied depending on the epididymal segment – from high in segment II of the caput to completely absent in the corpus epididymis. The presence of a higher number of clear cells in the cauda versus the caput epididymis [68], could explain the difference in staining pattern seen in these regions. Clear cells are known to play a role in luminal acidification, as they express H+V-ATPase and are involved in active proton secretion [69]. This expression pattern was surprisingly similar to what was reported in the gastric mucosa where TSPO was strongly expressed in the proton-secreting parietal cells [70]. However, functional studies using pharmacological TSPO modulators did not reveal a direct relationship between TSPO and proton secretion, but rather showed an effect on chloride secretion [70]. Although not directly demonstrated in the epididymis, chloride ions in most cases accompany the protons in the acidification of luminal compartments [71]. Therefore, there could be a function for TSPO in regulating the luminal pH in the epididymis. In addition, there is evidence that steroid hormone biosynthesis occurs in the cells of the epididymal epithelium. In rams, this has been demonstrated *in vitro* in principal cell cultures from the epididymal epithelium [72]. In the mouse, receptors for luteinizing hormone (LH), that trigger testosterone production in Leydig cells have also been localized to principal cells of the epididymis [73]. In our observation, we did find TSPO expression in principal cells, albeit at different levels between segments, that could participate in steroidogenesis. Therefore, TSPO function could be distinct for these functional cell types in the epididymal epithelium.

### TSPO Expression in the Male Accessory Sex Glands

**Figure 7:**
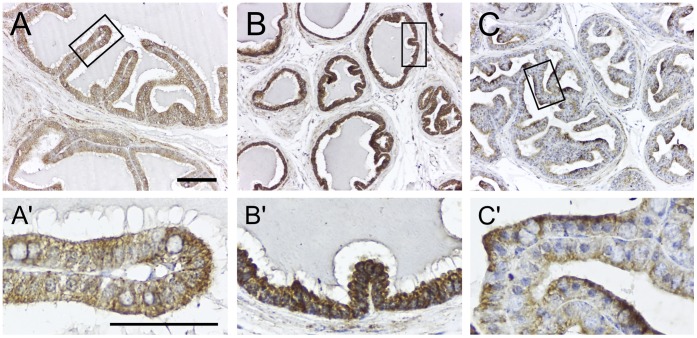
TSPO is expressed in the epithelium of the seminal vesicle and prostate. Immunohistochemical localization of TSPO in the seminal vesicle and prostate from an 8-week-old mouse. (A) Epithelium of the seminal vesicle showed weak expression of TSPO that was diffuse throughout the cytoplasm. (B) Epithelium of the ventral prostate showed strong expression of TSPO also distributed evenly in the cytoplasm. (C) Epithelium of the dorsolateral prostate showed a distinct apically polarized TSPO expression. Boxed regions in panels A, B and C are magnified in panels A’, B’ and C’. Scale bars 20 µm (Low magnification panel) and 10 µm (High magnification panel).

The seminal vesicle and prostate development have been largely studied as targets of androgen action in rodent models. Their secretory activity has been extensively investigated based on the composition and function of seminal fluid. We find that the seminal vesicle and prostate epithelia express TSPO (Fig. 7). In the ventral prostate, expression was higher compared to the dorsolateral prostate, in which TSPO was localized to the apical part of the cells. Based on work done in the rat, it is known that both seminal vesicle epithelia [74], and prostate epithelia [75] express LH receptor similar to testicular Leydig cells. However, there is no report of *de novo* steroidogenic function in these epithelia. Therefore, function of TSPO in these epithelial layers could be associated with secretory events linked to luminal pH modification [70], that may need to occur in these accessory sex glands. The paucity of data in this area also makes it plausible that *de novo* steroidogensis could be taking place in these accessory sex gland epithelia.

### TSPO Expression in the Adult and Pregnant Ovary

**Figure 8:**
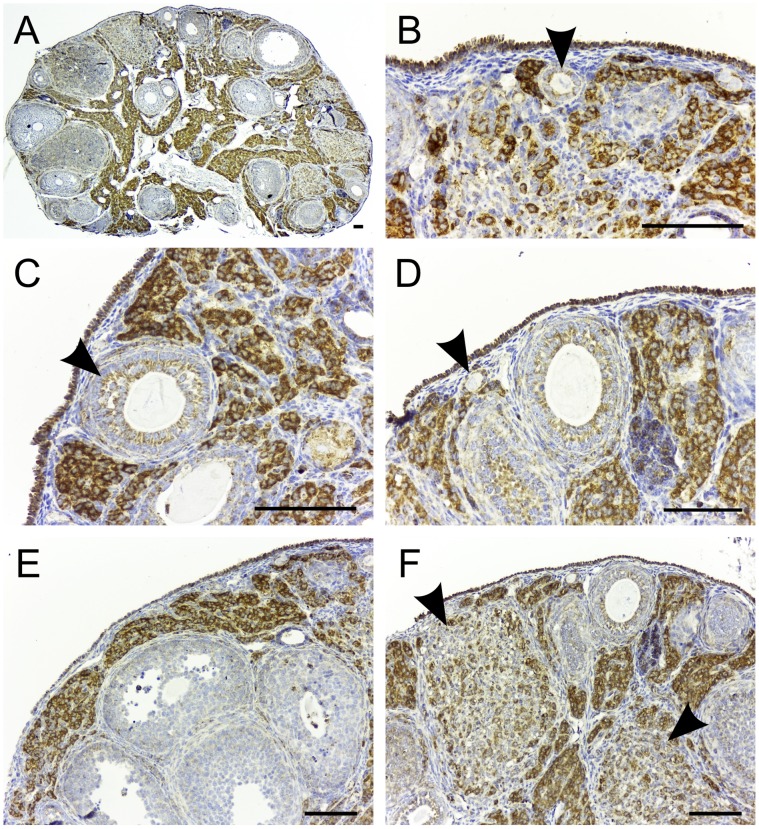
TSPO expression in the adult ovary is localized to the interstitial cells and granulosa cells. Immunohistochemical localization of TSPO in ovaries from an adult 8-week-old mouse. (A) The staining pattern for TSPO in a section dissecting the entire ovary. There was strong expression of TSPO observed in the interstitial cells. (B) TSPO expression was also strong in the ovarian surface epithelium in addition to the interstitial cells. Granulosa cells of a primary follicle (arrowhead) also showed TSPO expression. (C) Granulosa cells of a secondary follicle (arrowhead) expressed TSPO. Few theca cells around the follicle also showed weak expression of TSPO. (D) Squamous granulosa layer of a primordial follicle (arrowhead) showed TSPO expression. (E) Granulosa cells of most antral follicles show very weak to no expression of TSPO. (F) Regressing corpora lutea (arrowheads) also contained cells that show strong expression of TSPO. Scale bars 20 µm.

Integrated with its function, the ovary produces two major steroid hormones: estrogen during the follicular phase and progesterone during the luteal phase of the ovarian cycle. Studies using radiolabeled [^3^H]PK11195 showed rough localization of TSPO to the regions of interstitial cells, corpus luteum and follicles [76]. In this study, we observed high expression of TSPO in the interstitial cells of the ovary (Fig. 8). A weaker expression of TSPO was observed in the granulosa cells surrounding the developing follicles in the primordial, primary, secondary and tertiary stages. In antral follicles, TSPO expression in granulosa cells was heterogeneous. This could be due to the heterogeneity in steroidogenic potential of granulosa cells [77], that can correlate with TSPO expression. Theca cells surrounding these follicles also showed weak expression of TSPO. Based on the conserved two-cell, two-gonadotropin concept, theca and granulosa cells respond to LH and FSH to produce androstenedione and estrogen respectively [78], [79]. Ovarian interstitial cells also respond to LH and produce androstenedione [80]. Androstenedione from both theca and interstitial cells serve as a substrate for estrogen biosynthesis by the granulosa cell layers. Ovarian steroidogenesis and its regulation are well defined for these three cell types in the ovary (reviewed in [81]). Underscoring the potential cooperative role for TSPO in steroidogenic function, a pattern for StAR expression similar to our observation on TSPO has been reported for the rat ovary [82].

**Figure 9:**
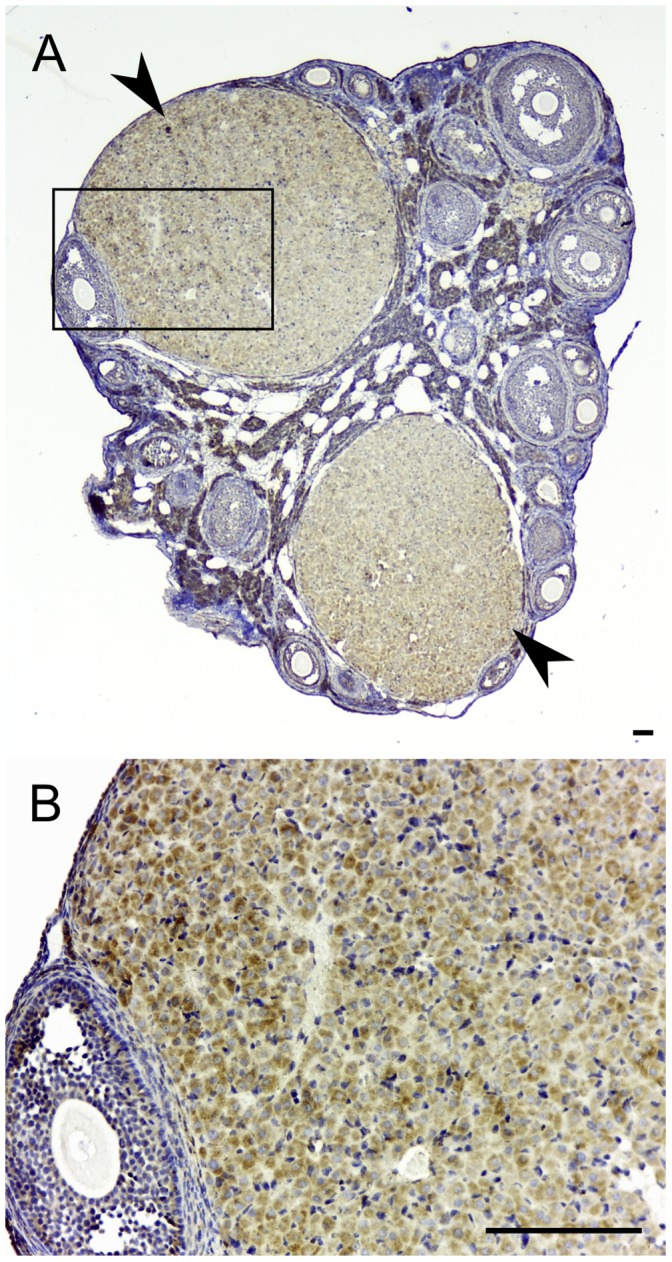
TSPO is expressed in the active corpus luteum. Immunohistochemical localization of TSPO in an ovary from a 14.5-day post coitus pregnant mouse. (A) The staining pattern for TSPO in a section dissecting the entire ovary. There was strong expression of TSPO in the two corpora lutea (arrowheads). (B) Higher magnification image of the boxed region in panel A showing variable levels of TSPO expression between different cells that form the corpus luteum. Both large and small luteal cells of the corpus luteum showed expression of TSPO. Scale bars 20 µm.

Estrogen production in the ovulating follicles is terminated by the LH surge transforming their theca and granulosa cells to corpora lutea that produce progesterone. In the pregnant ovary, we found that corpora lutea express TSPO (Fig. 9), at a level higher than that seen in theca and granulosa cells of the antral follicles. TSPO expression was seen in both large and small luteal cells, both capable of making progesterone albeit at different levels [83].

In addition to the steroidogenic cells, prominent TSPO expression was also distinctly present in the ovarian surface epithelium/germinal epithelium (Fig. 8). These squamous to cuboidal mesothelial cells with microvilli like projections that form the ovarian surface epithelium have been traced to form the embryonic origin of granulosa cells [84]. In pathologies, it has been suggested that these cells could be a cause for ovarian tumors [85] and they also believed to contain a population of germline stem cells for postnatal follicular renewal [86]. There is no known steroidogenic or secretory function for the germinal epithelium. Therefore, the integral activity for TSPO in this cell type remains to be investigated.

### TSPO Expression in the Developing Ovary

**Figure 10:**
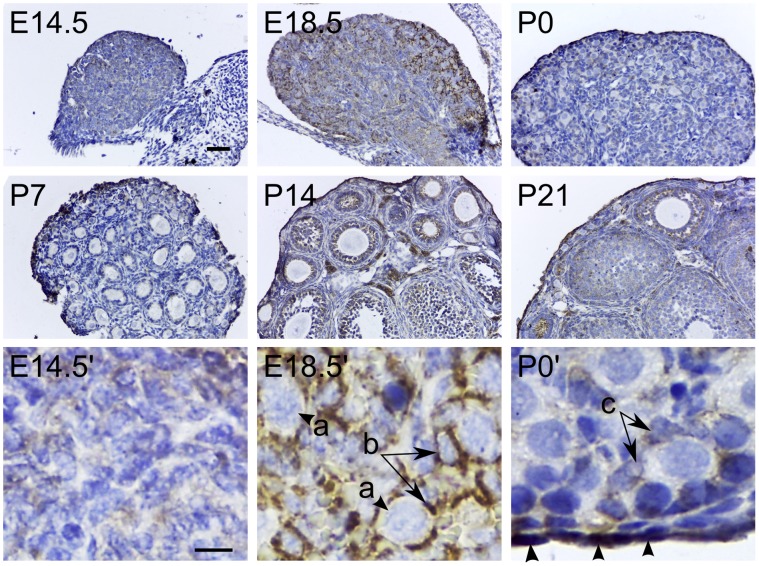
Developmental expression of TSPO in the ovary. Immunohistochemical localization of TSPO in embryonic (E14.5 and E18.5), and early postnatal stages (P0, P7, P14 and P21) of the developing ovary. At stages E14.5 and E18.5, TSPO was weak and appeared diffusely expressed in most cells forming the ovarian structure. Expression levels were higher at E18.5 compared to E14.5. Higher magnification images showed specific expression in the epithelial pregranulosa cells associated with the ovarian cysts (E14.5′ and E18.5′; a – progenitor in a germline cyst; b – pregranulosa cells). Compared to E18.5, TSPO expression at P0 was lower in the pregranulosa layer (P0′; c – pregranulosa cells) but there was strong expression in the germinal epithelium. Starting at P7, TSPO expression was observed in few clusters of interstitial cells that were very prominent in both numbers and expression at P14 and P21. TSPO expression pattern at P21 closely resembled that observed in the adult ovary. Scale bars 20 µm.

In premeiotic germ cell development, cysts due to incomplete cytokinesis arise from division of progenitor cells that subsequently enter meiosis [87]. In the E14.5 ovary, these cysts are at prophase I of meiosis. We find that TSPO expression is weak in the E14.5 ovary and does not appear to be associated with the germ line cysts (Fig. 10). This is supported by observations at E18.5, when TSPO expression is prominent in the pre-granulosa cells associated with the germ line cysts. At birth (P0), expression of TSPO persisted in squamous granulosa cells surrounding the primordial follicles. From E18.5 to P0, there was a progressive increase in expression of TSPO in the ovarian surface epithelium that persisted through adulthood. From P7, TSPO expression was seen in clusters of interstitial cells that progressively increased in numbers through P14 and P21. Expression level at P21 closely resembled findings reported for the adult ovary. There are no reports on the developmental expression of different steroid hydroxylases and/or StAR to compare with findings on TSPO expression in this study. A report on steroidogenic factor-1 (SF-1; a nuclear receptor that regulates steroid hydroxylases) and CYP11A1 transcripts in the developing ovary showed that neither SF-1 nor CYP11A1 were expressed from E13.5 to E16.5 [61]. However, weak expression of SF-1 was detected on E18.5. This suggests that after sex differentiation of the bipotential gonad, TSPO expression preceded the expression of enzymes required for the steroid hormone biosynthesis.

### TSPO Expression in the Uterus and Oviduct

**Figure 11:**
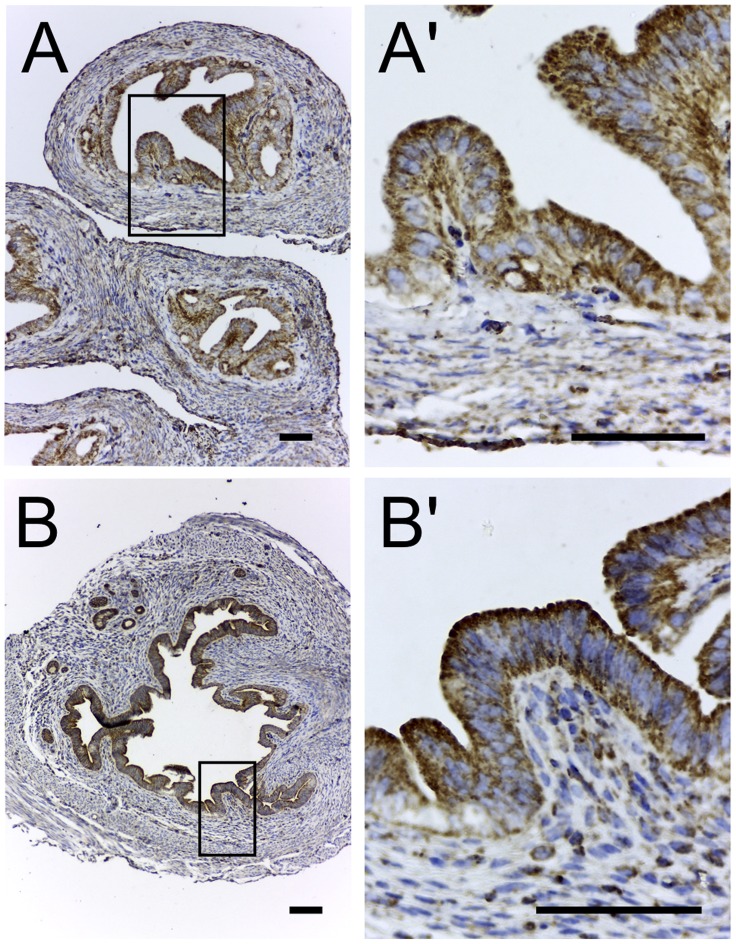
TSPO is expressed in the epithelium of the oviduct and uterus. Immunohistochemical localization of TSPO in the non-pregnant oviduct and uterus. (A) Epithelium of the oviduct showing strong expression of TSPO that was diffuse throughout the cytoplasm. (B) Epithelium of the uterus showing strong expression of TSPO that was distributed throughout the cytoplasm with specific enrichment towards the apical regions of the cells. Boxed regions in panels A and B are magnified in panels A’ and B’. Scale bars 20 µm.

In both the non-pregnant oviduct and uterus TSPO expression was present in the epithelial layer lining the lumen, along with a few scattered cells expressing TSPO in the submucosa/stroma (Fig. 11). This expression pattern was consistent and did not appear to change with either the different stages of the estrous cycle or the different regions within the uterus and oviduct. There is evidence for *de novo* estrogen production by the stromal cells of the uterine endometrium [88], but not the epithelium. Moreover, there are no reports on steroidogenic activity in the oviduct. Therefore, the function for TSPO in these luminal epithelia could be associated with secretory activity similar to chloride secretion and luminal acidification as seen in the gastric mucosa [70].

## Conclusions

TSPO is a protein that shows high sequence conservation from bacteria to mammals [89]. The well-studied function for TSPO in steroid hormone biosynthesis explains several of the physiological and pathological observations in different organ systems [90]. Equally, there are strong data suggesting multiple alternate functions for this protein [7]. Even in steroidogenic cells, it is clear that regulation of TSPO function can be complex. For example, its endogenous ligand acyl-CoA-binding protein (ACBP)/diazepam binding inhibitor was reported to induce steroidogenesis in adrenocortical cells *in vitro* [91]. However, mice deficient in ACBP do not have problems in steroid hormone biosynthesis [92], suggesting that multiple secondary levels of functional regulation could exist for TSPO.

At the core level of transcriptional regulation, effects of several TSPO gene upstream-binding elements (Sp1/Sp3, AP1, Ets and SINE B2) have been examined [26], [27], [28]. However, none of them completely explain the precise cell type-specific TSPO expression patterns seen in different tissues. Moreover, the basis of pathological upregulation of TSPO as seen in cancer cells remains to be established. These points suggest that regulation of this conserved gene is fairly complex and may differ based on the functional cell type.

In conclusion, this study presents several novel findings regarding TSPO localization in both the male and female reproductive system. There was clear indication of the multifunctional nature of TSPO evident from its specific expression in both steroidogenic and select non-steroidogenic cell types. These expression patterns in reproductive tissues will provide a functional reference for further studies to fully understand different TSPO functions.
